# Supplementary material for: Extracellular vesicles from microglial cells activated by abnormal heparan sulfate oligosaccharides from Sanfilippo patients impair neuronal dendritic arborization
Source: Mol Med. 2024 Nov 4;30:197. doi: 10.1186/s10020-024-00953-1 (PMC11536927; doi:10.1186/s10020-024-00953-1)
Supplement: Supplementary file 1 — Additional file 1. [file 10020_2024_953_MOESM1_ESM.pdf]

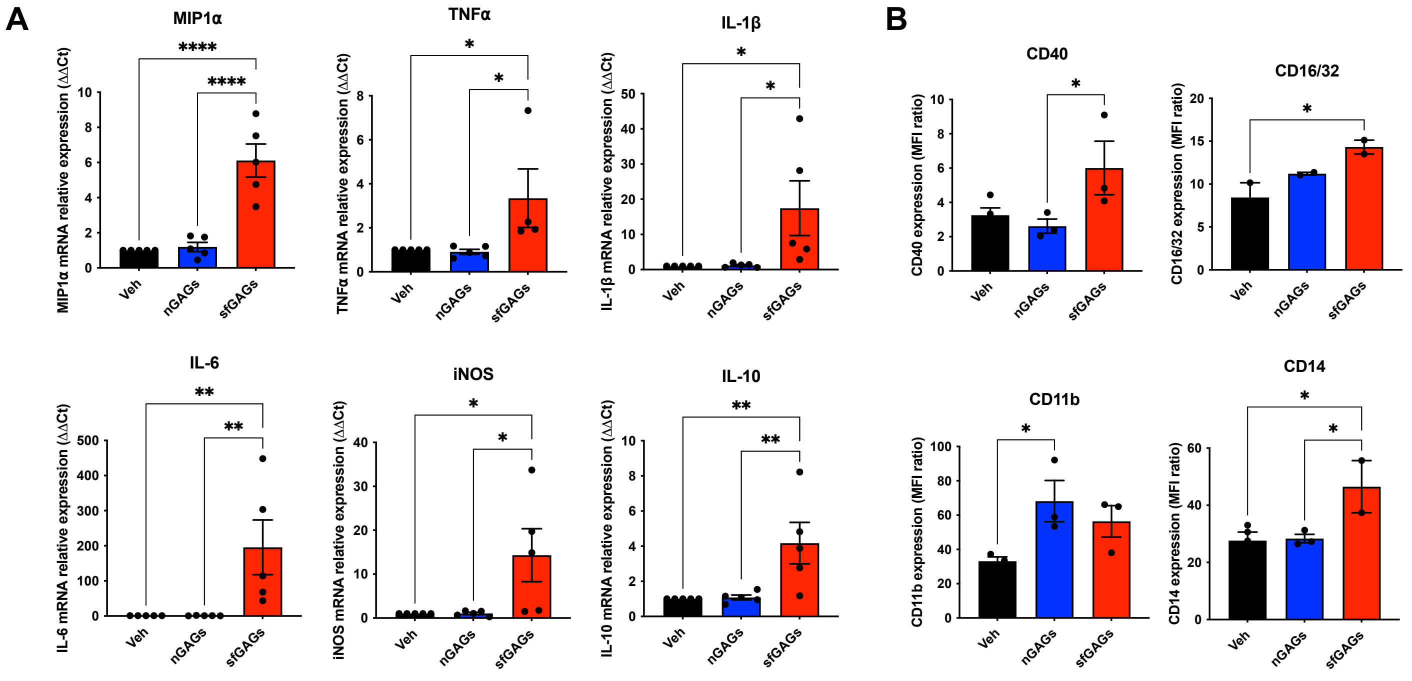

Fig. S1. Pro-inflammatory activation of BV-2 cells by GAGs extracted from urine of patients with Sanfilippo syndrome

(A) An RT-qPCR analysis confirmed higher mRNA expression levels for MIP1α (p=0.0003), IL-6 (p=0.0186), IL-1β (p=0.0335), TNFα (p=0.0643), iNOS (p=0.0291), and IL-10 (p=0.0137) in BV-2 cells treated with Sanfilippo GAGs (sfGAGs). **(B)** Flow cytometry analysis revealed higher protein expression levels for the myeloid surface markers CD16/32 (p=0.0322), CD11b (p=0.0314) and CD14 (p=0.0199) in BV-2 + sfGAGs. **(A-B)** BV-2 cells treated with sfGAGs (in red) were compared with BV-2 cells treated with normal GAGs (nGAGs, in blue) or vehicle only (Veh, in black) in bar plots. Unpaired t-test, n=2-5 biological replicates (each point represents 1 n), one-tailed p-value, ns = $p > 0.05$; * = $p \leq 0.05$; ** = $p \leq 0.01$; *** = $p \leq 0.001$.

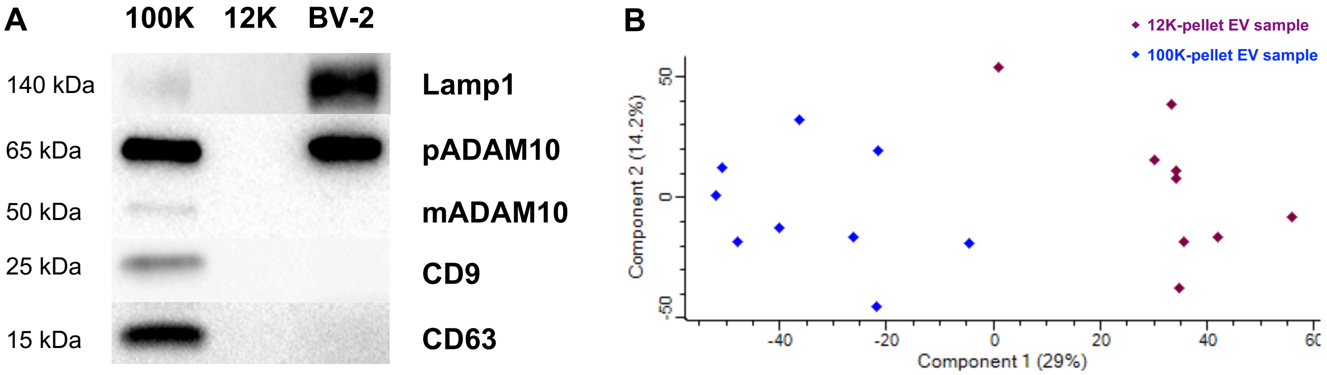

Fig S2. Quality check on proteins from large and small EVs.

(A) Western blots performed with Veh-100k EVs (100k), Veh-12k EVs (12k) and BV-2 cell lysates (BV-2) revealed the enrichment of specific EV protein markers CD9, CD63, pADAM10 and LAMP1 in Veh-100k EVs. **(B)** The results of a principal component analysis of the EV sample quality check, showing that the 12k-EV population (in brown) and the 100k-EV population (in blue) clustered together.

A

Sample	RNA concentration (ng/µL)
nGAGs-12k_1	1.92
nGAGs-12k_2	1.01
nGAGs-12k_3	7.30
sfGAGs-12k_1	2.49
sfGAGs-12k_2	5.58
sfGAGs-12k_3	6.02
nGAGs-100k_1	9.00
nGAGs-100k_2	5.08
nGAGs-100k_3	21.20
sfGAGs-100k_1	10.24
sfGAGs-100k_2	3.79
sfGAGs-100k_3	11.88

B

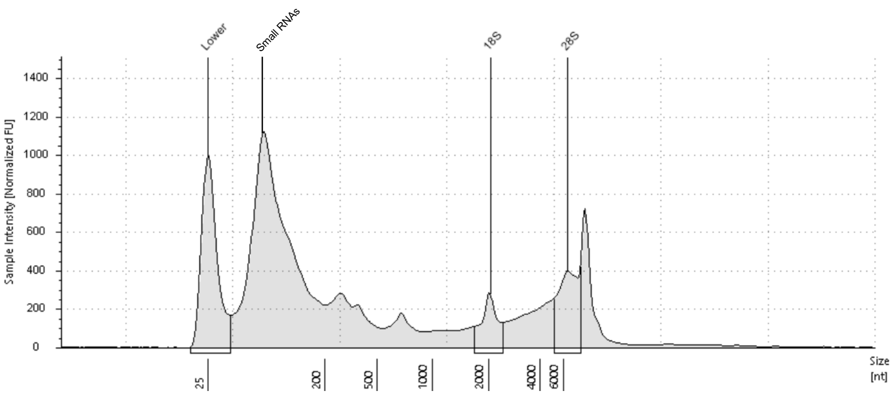

Fig. S3. Quality check of RNA samples from large and small EVs.

(A) Before sequencing, total RNA was quantified using a Qubit Fluorometer (Illumina QIAseq). **(B)** A representative total RNA electrophoresis profile evaluated on an Agilent 4150 TapeStation, showing the presence of small RNAs. Peaks from left to right: lower marker, small RNAs, ribosomal 18S and 28S RNAs.

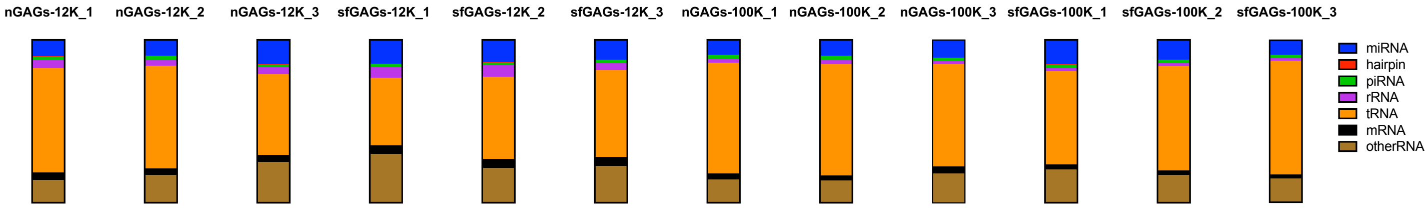

Fig. S4. Proportion of different types of RNA in EV samples. Transfer RNAs and miRNAs were the most frequent RNAs. The figure shows miRNAs (in blue), hairpins (in red), piRNAs (in green), rRNAs (in purple), tRNAs (in orange), mRNAs (in black), and other RNAs (in brown).

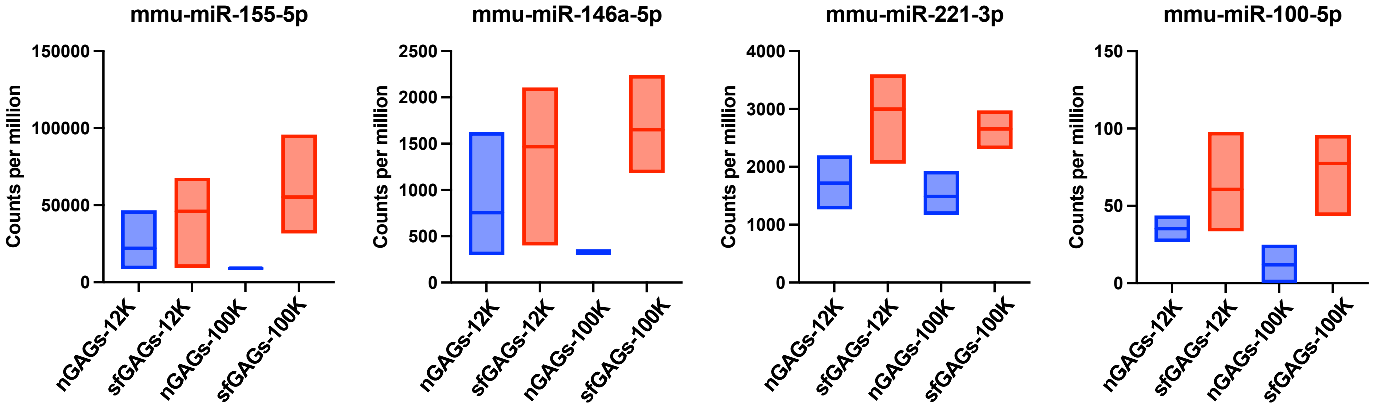

Fig. S5. Counts per million for the four miRNAs enriched in sfGAGs-EVs (represented as violin plots). EVs were collected from conditioned media from BV-2 cells treated with nGAGs (nGAGs-, blue), sfGAGs (sfGAGs-, red), and the RNA was extracted from the 12k pellet (-12k) or the 100k pellet (-100k). n=3 biological replicates (each bar of the violin plot represents 1 n).

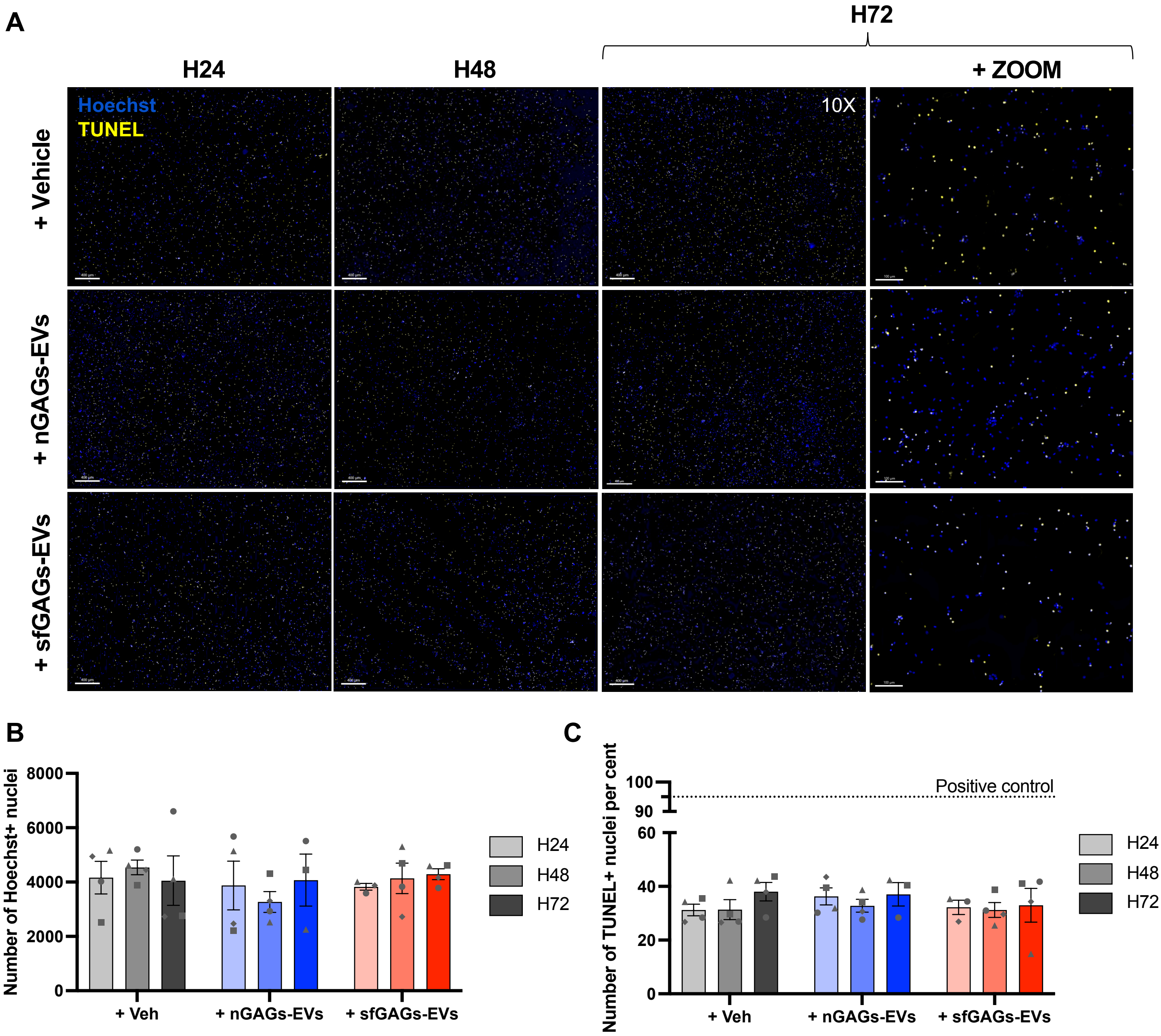

Fig. S6. DAPI staining and TUNEL assay results for neurons exposed to EVs

(A) Representative wide-field images of 25 square stitched mosaics (10X objective) of neurons treated with nGAGs-EVs, sfGAGs-EVs or with trehalose for 24 hours (H24), 48 hours (H48) or 72 hours (H72). The nuclei were stained with Hoechst 33342 reagent (in cyan) or Alexa Fluor^{TM} 488 dye (in yellow) when combined with a TUNEL assay. Scale bars = 400 µm for the main images and 100 µm for insets. **(B)** Exposure to EVs did not lead to a decrease in the number of Hoechst+ nuclei. **(C)** Quantification of colocalized TUNEL+ and Hoechst+ nuclei showed that EVs do not have an apoptotic effect on neurons. **(B, C)** Neurons treated with trehalose (+ vehicle, grey), nGAGs-EVs (+ nGAGs-EVs, blue) or sfGAGs-EVs (+ sfGAGs-EVs, red) after 24 hours (H24, in light grey), 48 hours (H48, in medium grey) or 72 hours (H72, in dark grey) of treatment. Two-way ANOVA, n=4 (each point represents 1 n).
