# Supplementary material for: Extracellular vesicles from microglial cells activated by abnormal heparan sulfate oligosaccharides from Sanfilippo patients impair neuronal dendritic arborization
Source: Mol Med. 2024 Nov 4;30:197. doi: 10.1186/s10020-024-00953-1 (PMC11536927; doi:10.1186/s10020-024-00953-1)
Supplement: Supplementary file 2 — Additional file 2. [file 10020_2024_953_MOESM2_ESM.pdf]

Gene names	Protein distribution in pellets	Uniprot IDs	Protein names
**Actn4**	12K	P57780	Alpha-actinin-4
**Hsp90b1**	12K	P08113	Endoplasmin
**Hspa1a; Hspa1b**	12K	Q61696;P17879	Heat shock 70 kDa protein 1A;Heat shock 70 kDa protein 1B
**Immt**	12K	Q8CAQ8	MICOS complex subunit Mic60
**Abcc1**	Both	O35379	Multidrug resistance-associated protein 1
**Arrdc1**	Both	Q99KN1	Arrestin domain-containing protein 1
**Bsg**	Both	P18572	Basigin
**CD63**	Both	P41731	CD63 antigen
**CD82**	Both	P40237	CD82 antigen
**CD9**	Both	P40240	CD9 antigen
**Flot1**	Both	O08917	Flotillin-1
**Hspa8**	Both	P63017	Heat shock cognate 71 kDa protein
**Hspg2**	Both	Q05793	Basement membrane-specific heparan sulfate proteoglycan core protein;Endorepellin;LG3 peptide
**Itga4**	Both	Q00651	Integrin alpha-4
**Itga5**	Both	P11688	Integrin alpha-5;Integrin alpha-5 heavy chain;Integrin alpha-5 light chain
**Itga6**	Both	Q61739	Integrin alpha-6;Integrin alpha-6 heavy chain;Integrin alpha-6 light chain
**Itgal**	Both	P24063	Integrin alpha-L
**Itgam**	Both	P05555	Integrin alpha-M
**Itgav**	Both	P43406	Integrin alpha-V;Integrin alpha-V heavy chain;Integrin alpha-V light chain
**Itgax**	Both	Q9QXH4	Integrin alpha-X
**Itgb1**	Both	P09055	Integrin beta-1
**Itgb2**	Both	P11835	Integrin beta-2
**Itgb7**	Both	P26011	Integrin beta-7
**Lamp1**	Both	P11438	Lysosome-associated membrane glycoprotein 1
**Rhoa**	Both	Q9QUI0	Transforming protein RhoA
**Sdc1**	Both	P18828	Syndecan-1
**Sdc3**	Both	Q64519	Syndecan-3
**Sdc4**	Both	O35988	Syndecan-4
**Sdcbp**	Both	O08992	Syntenin-1
**Tfrc**	Both	Q62351	Transferrin receptor protein 1
**Vps4b**	Both	P46467	Vacuolar protein sorting-associated protein 4B
**Anxa2**	100K	P07356	Annexin A2
**Anxa3**	100K	O35639	Annexin A3
**Anxa5**	100K	P48036	Annexin A5
**Anxa8**	100K	O35640	Annexin A8
**Adam10**	100K	O35598	Disintegrin and metalloproteinase domain-containing protein 10
**Anxa1**	100K	P10107	Annexin A1
**Anxa11**	100K	P97384	Annexin A11
**Anxa4**	100K	P97429	Annexin A4
**Anxa6**	100K	P14824	Annexin A6
**Anxa7**	100K	Q07076	Annexin A7
**CD81**	100K	P35762	CD81 antigen
**Pdcd6ip**	100K	Q9WU78	Programmed cell death 6-interacting protein
**Tsg101**	100K	Q61187	Tumor susceptibility gene 101 protein

Table S1. List of specific protein markers of large and small EVs, as identified by mass spectrometry.

Reads set	nGAGs-12k_1		nGAGs-12k_2		nGAGs-12k_3		sfGAGs-12k_1		sfGAGs-12k_2		sfGAGs-12k_3	
	Reads number	%	Reads number	%	Reads number	%	Reads number	%	Reads number	%	Reads number	%
**total_reads**	**14 171 376**	**100**	**14 028 393**	**100**	**12 142 987**	**100**	**14 542 659**	**100**	**11 742 528**	**100**	**14 622 539**	**100**
no_adapter_reads	661 606	5	921 381	7	1 195 919	10	750 957	5	840 160	7	747 963	5
too_short_reads	2 529 611	18	3 055 470	22	1 842 653	15	2 488 025	17	1 924 101	16	2 288 878	16
UMI_defective_reads	914 853	6	1 277 084	9	1 865 634	15	1 625 728	11	1 397 566	12	1 731 679	12
**Total reads - defaults**	**10 065 306**	**71**	**8 774 458**	**63**	**7 238 781**	**60**	**9 677 949**	**67**	**7 580 701**	**65**	**9 854 019**	**67**
miRNA_Reads	733 705	5	523 473	4	798 777	7	1 121 081	8	776 996	7	869 015	6
hairpin_Reads	11 993	0	14 180	0	14 881	0	14 318	0	12 703	0	13 329	0
piRNA_Reads	145 270	1	121 959	1	71 946	1	118 187	1	86 358	1	114 866	1
rRNA_Reads	366 310	3	215 689	2	234 357	2	505 560	3	404 048	3	326 975	2
tRNA_Reads	4 632 323	33	3 425 267	24	2 643 947	22	3 168 072	22	2 820 223	24	3 773 447	26
mRNA_Reads	328 920	2	225 739	2	233 529	2	378 021	3	316 174	3	381 509	3
otherRNA_Reads	1 002 687	7	925 160	7	1 328 657	11	2 286 748	16	1 192 253	10	1 605 922	11
notCharacterized_Mappable	848 410	6	948 710	7	591 278	5	669 948	5	617 793	5	806 159	6
notCharacterized_notMappable	1 995 688	14	2 374 281	17	1 321 409	11	1 416 014	10	1 354 153	12	1 962 797	13

Reads set	nGAGs-100k_1		nGAGs-100k_2		nGAGs-100k_3		sfGAGs-100k_1		sfGAGs-100k_2		sfGAGs-100k_3	
	Reads number	%	Reads number	%	Reads number	%	Reads number	%	Reads number	%	Reads number	%
**total_reads**	**11 868 744**	**100**	**13 006 860**	**100**	**13 624 495**	**100**	**13 000 906**	**100**	**10 788 895**	**100**	**14 283 442**	**100**
no_adapter_reads	657 906	6	851 701	7	630 224	5	1 034 491	8	767 790	7	689 372	5
too_short_reads	1 920 935	16	2 027 500	16	2 344 861	17	2 085 611	16	1 999 125	19	1 909 175	13
UMI_defective_reads	1 096 089	9	1 518 644	12	1 222 593	9	1 501 764	12	1 009 511	9	1 390 879	10
**Total reads - defaults**	**8 193 814**	**69**	**8 609 015**	**66**	**9 426 817**	**69**	**8 379 040**	**64**	**7 012 469**	**65**	**10 294 016**	**72**
miRNA_Reads	517 787	4	587 482	5	696 007	5	949 021	7	596 865	6	695 392	5
hairpin_Reads	13 336	0	15 300	0	12 592	0	16 669	0	10 947	0	16 699	0
piRNA_Reads	148 693	1	151 845	1	108 017	1	123 306	1	106 369	1	152 232	1
rRNA_Reads	113 033	1	161 104	1	138 852	1	129 807	1	92 205	1	121 951	1
tRNA_Reads	3 874 784	33	4 151 724	32	4 069 290	30	3 607 229	28	3 148 299	29	5 489 858	38
mRNA_Reads	192 852	2	188 884	1	266 619	2	187 639	1	133 068	1	176 971	1
otherRNA_Reads	811 183	7	829 882	6	1 167 023	9	1 294 313	10	846 707	8	1 143 937	8
notCharacterized_Mappable	706 579	6	743 437	6	839 704	6	654 517	5	620 655	6	753 382	5
notCharacterized_notMappable	1 815 567	15	1 779 357	14	2 128 713	16	1 416 539	11	1 457 354	14	1 743 594	12

Table S2. Number of reads and percentage of different types of RNA from each EV sample.

The data were produced by the Legacy Analysis Pipeline on the QIAGEN GeneGlobe portal.
